# Tracking the crystallization behavior of high-silica FAU during AEI-type zeolite synthesis using acid treated FAU-type zeolite†

**DOI:** 10.1039/d1ra03150c

**Published:** 2021-06-29

**Authors:** Yuki Sada, Anand Chokkalingam, Kenta Iyoki, Masato Yoshioka, Tomoya Ishikawa, Yusuke Naraki, Yutaka Yanaba, Hiroki Yamada, Koji Ohara, Tsuneji Sano, Tatsuya Okubo, Zhendong Liu, Toru Wakihara

**Affiliations:** Department of Chemical System Engineering, The University of Tokyo 7-3-1 Hongo Bunkyo-ku Tokyo 113-8656 Japan liuzd@chemsys.t.u-tokyo.ac.jp wakihara@chemsys.t.u-tokyo.ac.jp; Inorganic Materials Research Laboratory, Tosoh Corporation 4560 Kaiseicho, Shunan Yamaguchi 746-8501 Japan; Institute of Industrial Science, The University of Tokyo 4-6-1 Komaba Meguro-ku Tokyo 153-8505 Japan; JASRI 1-1-1 Kouto, Sayo-cho Sayo-gun Hyogo 679-5198 Japan; Institute of Engineering Innovation, School of Engineering, The University of Tokyo 2-11-16 Yayoi Bunkyo-ku Tokyo 113-8656 Japan

## Abstract

During AEI zeolite synthesis using acid treated FAU (AcT-FAU), we found the recrystallization of high-silica FAU with high crystallinity and Si/Al ratio of 6.1 using *N*,*N*-dimethyl-3,5-dimethylpiperidinium hydroxide (DMDMPOH) after 2 h, followed by the crystallization of AEI *via* FAU-to-AEI interzeolite conversion at a longer synthesis time. In order to understand the formation mechanism of high-silica FAU and generalize its direct synthesis, we have investigated this synthesis process. An analysis of the short-range structure of AcT-FAU revealed that it has an ordered aluminosilicate structure having a large fraction of 4-rings despite its low crystallinity. The changes in the composition of the products obtained at different synthesis times suggested that DMDMP^+^ plays a certain role in the stabilization of the FAU zeolite framework. Moreover, the results of thermogravimetric analysis showed that the thermal stability of DMDMP^+^ changed with the zeolite conversion. To the best of our knowledge, this is the first study to clarify the structure-directing effect of DMDMP^+^ on FAU zeolite formation.

## Abstract 2

A high-silica FAU was obtained during FAU-to-AEI interzeolite conversion using acid treated FAU.

## Graphical abstract

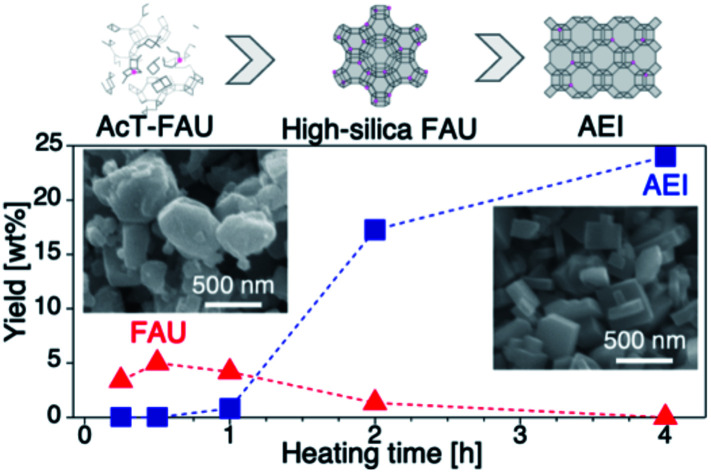

## Introduction

Zeolites have been widely used in the petrochemical industry and other chemical industries for more than half a century. They are crystalline microporous materials with various framework structures and have excellent characteristics such as adsorption, ion-exchange and molecular sieving. In particular, faujasite-type zeolite (FAU) is known as a fluid catalytic cracking and hydrocracking catalyst.^1,2^ When used as a catalyst, FAU with a Si/Al ratio of more than 5 is generally preferable to achieve high structural stability and catalytic activity.^3,4^ The high-silica FAU has been prepared by post-synthesis treatments such as high-temperature steaming,^5,6^ hydrothermal,^1^ and chemical treatments.^7^ That is because FAU with a Si/Al ratio of more than 3 cannot be obtained directly through a conventional hydrothermal synthesis without organic structure-directing agents (OSDAs)^8–10^ although the OSDA-free synthesis of FAU with a Si/Al ratio of about 3.2 by a hydroxyl radical assisted route has been recently reported.^11^ In previous studies, it was also reported that OSDA-free aluminosilicate FAU is thermodynamically stable in a low Si/Al ratio with a range approximately 2–3.^10,12^ Therefore, the FAU framework becomes energetically less stable with increasing the Si/Al ratio. This thermodynamical instability explains the difficulty in directly synthesizing high-silica FAU.

In contrast to the OSDA-free synthesis, FAU with a Si/Al ratio of approximately 4 can be synthesized with cyclic ether organic compounds such as 15-crown-5 and 18-crown-6,^13–15^ which stabilize the FAU zeolite framework due to the formation of bulky organic–inorganic complexes in the pore structure. Moreover, high-silica FAU with a Si/Al ratio as high as 7.8 has recently been synthesized using quaternary-ammonium-based OSDAs.^16^ In the research, the starting nuclei solution containing a tetraethylammonium hydroxide is heated to prepare a nanoparticulate FAU zeolite precursor. After additionally heating the FAU nuclei solution mixed with a bulky OSDA such as tetrabutylammonium hydroxide, which serves as a filler and alkaline source, high-silica FAU is obtained. Surely, this method is a novel synthesis method based on a systematic strategy. However, the issue remains that the procedure is complicated, with a total synthesis time of 7 days and has more than three heating steps including the aging process. Hence, it is still necessary to improve the direct synthesis method for high-silica FAU.

To solve this problem, we need to reconsider the roles of the OSDA in stabilizing the zeolite framework structure and that of silicon and aluminium sources as the starting materials. Recently, we have been focusing on AEI zeolite synthesis from FAU. Some studies on AEI zeolite synthesis^17–20^ have been reported in recent years as the zeolite is expected to be a novel NH_3_-SCR catalyst.^21–24^ Compared to recent studies, however, we have worked^25^ with the originality of AEI zeolite synthesis using various acid treated FAUs (AcT-FAUs) combined with *N*,*N*-dimethyl-3,5-diethylpiperidinium hydroxide (DMDMPOH), which is known as a typical OSDA for AEI zeolite synthesis.^17–20^ Through that research, we confirmed a unique crystallization behavior, that is, the recrystallization of FAU from AcT-FAU followed by AEI zeolite crystallization.^25^ In this study, we focus on the recrystallization behaviour of AcT-FAU. Surprisingly, the obtained FAU was found to be so high in the Si/Al ratio, which could not be easily achieved by a direct synthesis from the amorphous matters. Herein, we report on the mechanism of the recrystallization process of high-silica FAU from AcT-FAU, focusing on three points: (i) characterization of AcT-FAU, (ii) characterization of high-silica FAU, and (iii) tracking of the recrystallization process, which provides further information concerning not only the properties of the starting material or products, but also the role of SDAs such as organic or inorganic cations. These will help us to understand the effect of synthesis parameters as well as OSDA for the novel synthesis of high-silica FAU.

## Methods

### Preparation of the acid treated FAU zeolite

The starting H-type FAU (HSZ-320HOA, Si/Al = 2.8) was provided by Tosoh Corporation. Sulfuric acid (97%) was purchased from FUJIFILM Wako Pure Chemical Corporation. 0.70 M H_2_SO_4_ aq. was prepared by diluting the sulfuric acid.

Then, 5.0 g of H-type FAU and 50 g of 0.70 M H_2_SO_4_ aq. were mixed and heated at 75 °C for 4 h under stirring. The solid product was recovered by filtration, thoroughly washed using deionized water, and dried at 80 °C, thereby yielding the acid treated FAU (AcT-FAU).

### Synthesis of the zeolites

Sodium hydroxide (97%) was purchased from FUJIFILM Wako Pure Chemical Corporation. *N*,*N*-Dimethyl-3,5-dimethylpiperidinium hydroxide (DMDMPOH) was provided by Tosoh Corporation, where it was prepared by the ion-exchange of *N*,*N*-dimethyl-3,5-dimethylpiperidinium bromide using an ion-exchange resin, DIAION™ SA10AOH (Mitsubishi Chemical Corporation).

4.4 g of DMDMPOH was mixed with 2.0 g of sodium hydroxide aqueous solution, and then 3.0 g of AcT-FAU was dispersed in the mixed solution. This starting mixture was heated at 180 °C in a Teflon®-lined reactor sealed in a stainless-steel autoclave, or in a stainless-steel tube reactor at a static condition for 15 min to 10 h. It was synthesized at a scale of one-sixth when a stainless-steel tube reactor was used. The solid product was recovered by filtration, thoroughly washed using deionized water, and dried at 80 °C.

### Characterization

Powder X-ray diffraction (XRD) patterns were recorded on a diffractometer with CuKα radiation (*λ* = 1.54056 Å, Rigaku Ultima IV, 40 kV, 40 mA) at a scanning rate of 12° min^−1^ over a range of 3–50°. Thermogravimetric analyses were performed on a PU 4K (Rigaku) with a heating rate of 10 °C min^−1^, using a mixture of 10% O_2_ and 90% N_2_ as a carrier gas with a flow rate of 0.20 L min^−1^. The samples were dissolved in hydrofluoric acid and characterized by an inductively coupled plasma-atomic emission spectrometer (ICP-AES, iCAP-6300, Thermo Scientific). The solid-state ^29^Si magic-angle spinning nuclear magnetic resonance (MAS NMR) spectra were recorded on a JNM-ECA 500 (JEOL) at 99.4 MHz with a pulse length (π/2) of 5.0 μs, relaxation delay of 60 s, and spinning frequency of 10 kHz. The ^27^Al MAS NMR spectra were recorded at 130.33 MHz with a π/2 pulse length of 3.2 μs, a recycle delay of 5 s, and a spinning frequency of 14 kHz. Then, the peak areas of the ^29^Si MAS NMR spectra were deconvoluted with Lorentzian functions using the dmfit software. The ^13^C cross-polarization/magic-angle spinning (CP/MAS) NMR spectra were recorded at 125.76 MHz with a relaxation delay of 1.2 s, a contact time of 1 ms, and a spinning frequency of 10 kHz. The surfaces, morphologies, and sections of the samples were observed using a field-emission scanning electron microscope (JSM-7400F, JEOL). The sliced samples were prepared by embedding zeolite in a thermosetting resin and cutting out a cross section using a cross-section polisher (SM-09010, JEOL). UV Raman spectra were measured by a micro laser Raman spectroscopy (LabRAM HR Evolution, HORIBA) with a spectral resolution of 0.2 cm^−1^, in which the laser line at 325 nm of a He/Cd laser was used as an exciting source with an output of 50 mW, and the laser power on the sample was approximately 6.0 mW. Nitrogen adsorption–desorption isotherms were obtained on an Anton Paar NOVAtouch instrument at the liquid nitrogen temperature after an outgas pretreatment at 350 °C for 5 h under vacuum. Then, the high-silica FAU calcined at 550 °C for 5 h in a muffle furnace was used. High-energy X-ray total scattering (HEXTS) measurements were conducted at the BL04B2 high-energy XRD beamline (SPring-8, Japan). The powder sample was held in a quartz capillary tube (*φ*: 3.5 mm). Scattering patterns were measured at room temperature using a horizontal two-axis diffractometer under the vacuum condition. The energy of incident X-ray was 61.37 keV (*λ* = 0.202 Å) and the maximum *Q* (*Q* = 4π sin *θ*/*λ*), *Q*_max_, collected in this study was 25.8 Å^−1^ for zeolite samples. Furthermore, the obtained data were handled according to well-established analysis procedures,^26^ such as absorption, background, and Compton scattering corrections, and subsequently normalized to give a Faber–Ziman total structure factor *S*(*Q*).^27^ These *S*(*Q*)s were used to calculate the reduced pair distribution function (PDF), *G*(*r*), using the function
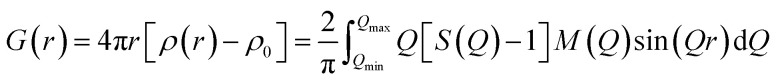

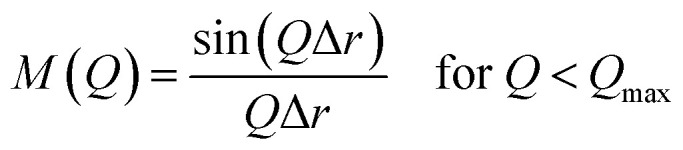
*M*(*Q*) = 0 for *Q* > *Q*_max_where *ρ* is the atomic number density and *M*(*Q*) is the window function developed by Lorch.^28^

## Results and discussion

### Preparation and characterization of the acid treated FAU with very low crystallinity as starting material

**Fig. 1:**
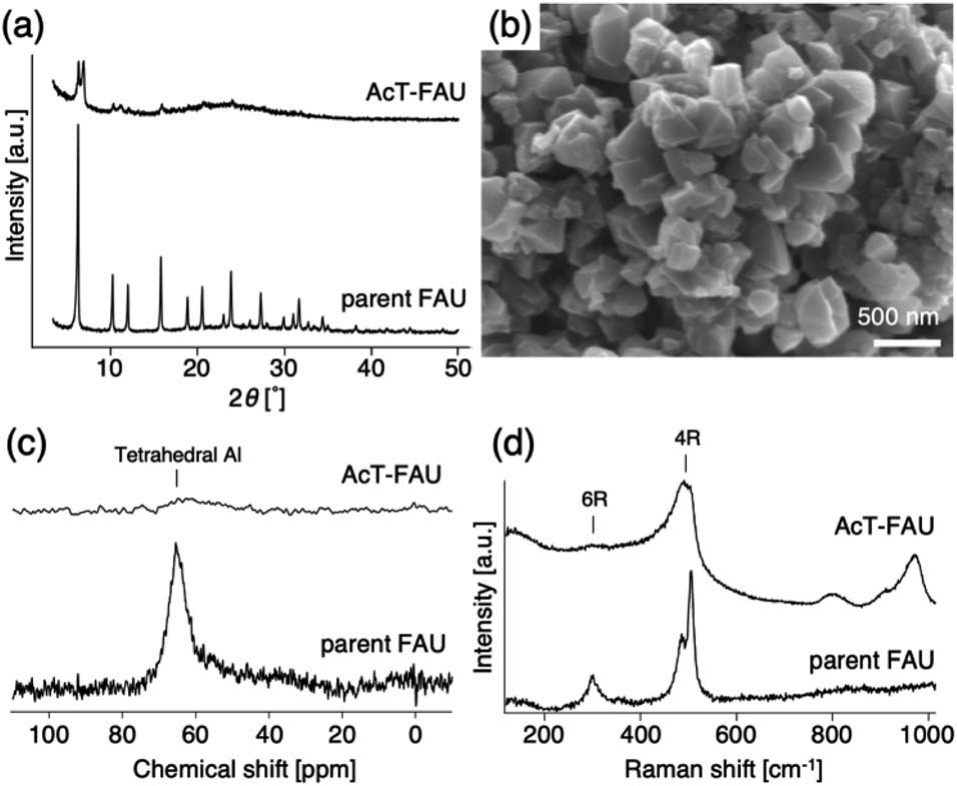
(a) XRD patterns of the parent FAU (Tosoh 320HOA) and acid treated FAU (AcT-FAU), (b) SEM image of the AcT-FAU, (c) ^27^Al MAS NMR spectra and (d) UV-Raman spectra of the parent FAU (Tosoh 320HOA) and AcT-FAU.

The XRD patterns of the parent FAU and AcT-FAU are shown in Fig. 1a. It was found that AcT-FAU possessed almost no crystallinity, although small peaks were observed on the low angle region. The Si/Al ratio of AcT-FAU measured by ICP-AES was 27.7. In addition, the yield of AcT-FAU was about 75 wt%. The SEM image shows that the particle size of the AcT-FAU was approximately 200–500 nm (Fig. 1b). Compared to the parent FAU (Fig. S1†), both the particle size and the morphology of AcT-FAU hardly changed. On the other hand, the results of the ^27^Al MAS NMR analysis (Fig. 1c) showed no peak due to the tetrahedral Al for AcT-FAU. This result indicates that the AcT-FAU was amorphized to a very low crystallinity, which was consistent with the XRD patterns. To compare the differences in the short-range structures, UV-Raman spectra of both the parent FAU and AcT-FAU were measured. As shown in Fig. 1d, microstructures such as 4- and 6-rings (4 and 6Rs) originating from the parent FAU changed significantly. In particular, the peaks at approximately 300 cm^−1^, attributed to the 6R^29–31^ decreased markedly and had significantly broadened. Additionally, the peaks at approximately 550 cm^−1^, attributed to 4R^29–31^ also decreased and broadened, but not as much as those of the 6R. This result suggests that the AcT-FAU still contains an ordered aluminosilicate structure with a large fraction of 4R.

### Recrystallization of high-silica FAU from the acid treated zeolite

**Fig. 2:**
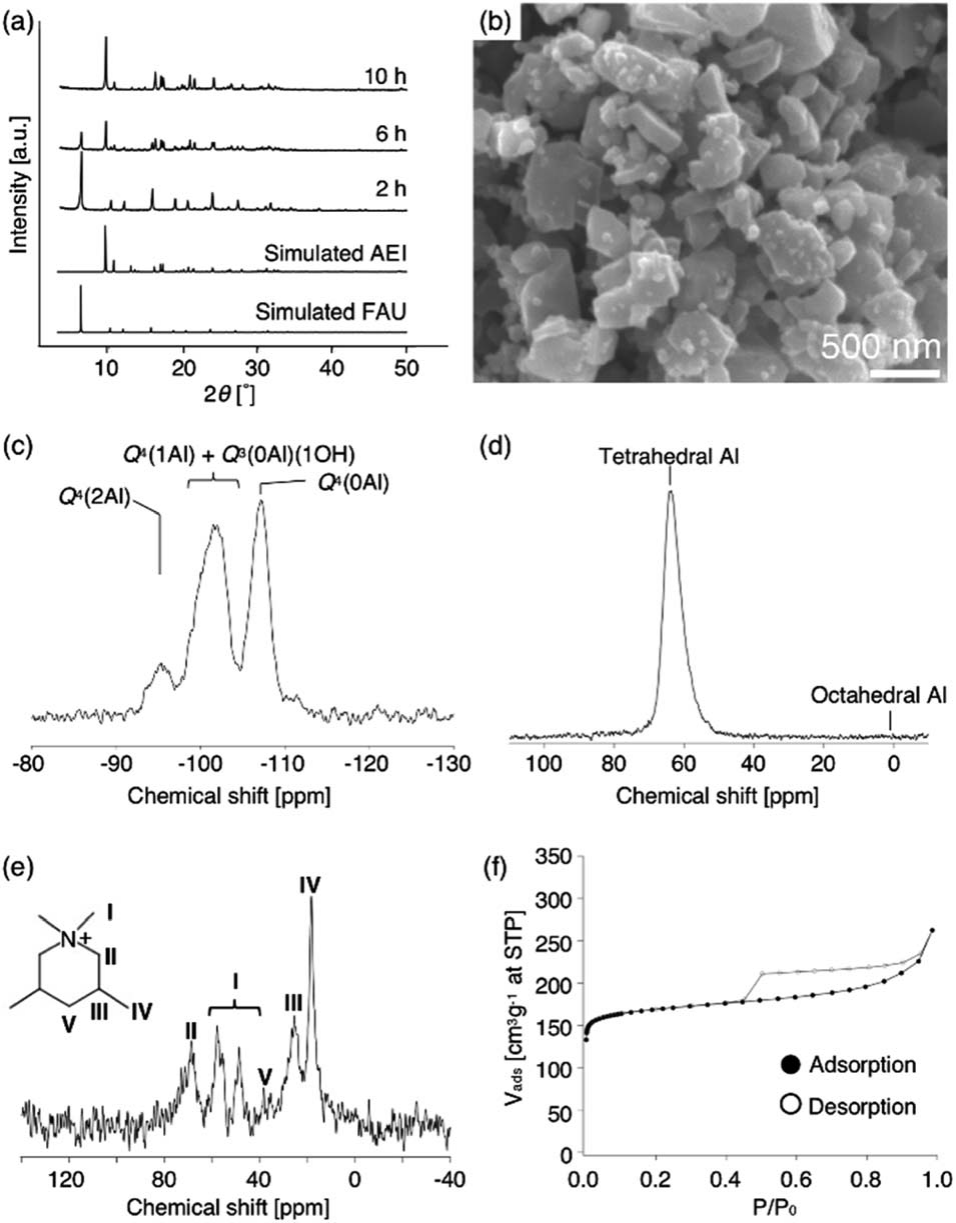
(a) XRD patterns of the products at 2, 6, and 10 h (as-made), (b) SEM image (as-made), (c) ^29^Si MAS NMR spectrum, (d) ^27^Al MAS NMR spectrum (as-made), (e) ^13^C CP/MAS NMR spectrum (as-made) and (f) N_2_ adsorption–desorption isotherms of high-silica FAU obtained from acid treated FAU at 2 h. Starting gel composition; 1.0 SiO_2_ : 0.018 Al_2_O_3_ : 0.15 Na_2_O : 0.20 DMDMPOH : 5.0 H_2_O.

Considering the previous studies that stated the FAU-to-AEI interzeolite conversion takes place using DMDMPOH as an OSDA, we tried to synthesize AEI from AcT-FAU at 180 °C using a starting reactant mixture with a chemical composition of 1.0 SiO_2_ : 0.018 Al_2_O_3_ : 0.15 Na_2_O : 0.20 DMDMPOH : 5.0 H_2_O. When the synthesis period was set for 10 h using a Teflon®-lined reactor sealed in a stainless-steel autoclave, pure AEI was obtained, which was similar to the previous FAU-to-AEI interzeolite conversion.^17–20^ In addition, surprisingly, we discovered that highly crystalline FAU was obtained after a 2 h synthesis during AEI zeolite synthesis, despite the use of the nearly amorphous AcT-FAU. The XRD patterns of the products (as-made) are shown in Fig. 2a, which indicates the formation of a pure FAU. The Si/Al ratio of the resulting FAU measured by ICP-AES was 6.1, which was much higher than that of the parent FAU. This means that the obtained FAU is not a remained parent FAU, indicating that understanding this phenomenon is important for revealing the formation mechanism of both of high-silica FAU and AEI. Considering the composition of AcT-FAU, the Si/Al ratio of the solid phase decreased significantly from 27.7 to 6.1. Moreover, based on the SEM images, it was found that a morphology of the obtained FAU was similar to that of the parent FAU and AcT-FAU (Fig. S1† and 1b), but there appeared to be a large variation in the particle size which was approximately 100–500 nm (as-made, Fig. 2b). For zeolites treated by the conventional recrystallization method using a silane coupling agent, the Si/Al ratio either increases or hardly changes due to defect healing. On the contrary, the Si/Al ratio of the solid phase after a synthesis decreases under most synthetic conditions in the interzeolite conversion method,^32–35^ which has unique synthesis conditions different from the conventional zeolite synthesis using amorphous matters. These results suggest that the resulting high-silica FAU was not formed from AcT-FAU by a simple recrystallization process with healing defects under hydrothermal condition; there should be another mechanism involved.

**Fig. 3:**
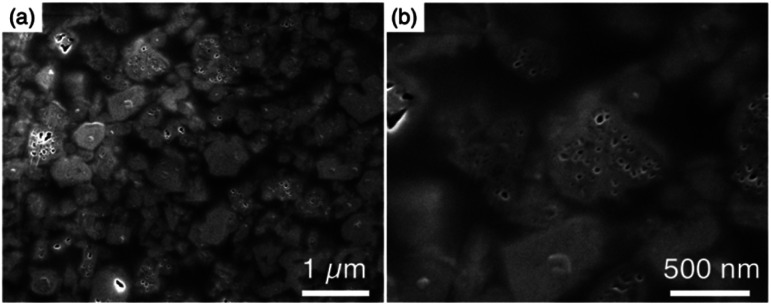
SEM images of sliced high-silica FAU (a) low magnification, and (b) high magnification.

To obtain further information concerning the formation process, we first characterized the obtained products. ^29^Si and ^27^Al MAS NMR spectra were obtained in order to confirm the chemical sates of the Si and Al atoms in the high-silica FAU (as-made, Fig. 2c and d). Three peaks were observed at −107, −104 to −98, and −95 ppm in the ^29^Si MAS NMR spectrum (Fig. 2c). The peaks at −107 ppm and −95 ppm were attributed to Q^4^(0Al) and Q^4^(2Al), respectively.^33,36^ In contrast, the peak at −104 to −98 ppm was broader and slightly asymmetric. Thus, the peak was assumed to be a superposition of Q^4^(1Al) and Q^3^(0Al)(1OH), based on a previous report.^16^ In addition, only a peak derived from tetrahedral Al was observed in the ^27^Al MAS NMR spectrum (Fig. 2d), indicating that there are no extra-framework Al species in the resultant high-silica FAU. The ^13^C CP/MAS NMR spectrum was also measured to identify the chemical structure of organic molecules present in zeolite pores (as-made, Fig. 2e). Typical peaks assigned to DMDMP^+^ cations were observed,^18^ indicating that the DMDMP^+^ cation is present and intact in the pores. Furthermore, the Brunauer–Emmett–Teller (BET) surface area and micropore volume of the high-silica FAU (calcined) calculated from the N_2_ adsorption isotherm were 502 m^2^ g^−1^ and 0.23 cm^3^ g^−1^, respectively. The values were slightly smaller compared to FAU obtained by a conventional direct synthesis, but without large difference. As shown in Fig. 2f, however, a large hysteresis loop in the adsorption–desorption isotherm of high-silica FAU was confirmed. Such a large hysteresis loop cannot be found in FAU-type zeolites that can be directly synthesized nor in high-silica FAU-type zeolites prepared by a conventional post-treatment.^1,5–7^ Typically, this hysteresis is classified as the H4 type hysteresis loop^37^ and derived from cavitation depending on the ink-bottle-shaped cavities covered with small pore openings. Particularly with zeolites, it is known that this hysteresis is confirmed due to the hollow architecture in the zeolite particles.^38^ Hence, we anticipated that this high-silica FAU would have mesoporous features inside the crystals. To confirm this possibility, cross-sectional SEM images were taken, which are shown in Fig. 3. Large voids inside the crystals are confirmed clearly in the high-silica FAU, which were not observed in the parent FAU (Fig. S2a†). In addition, the cross-sectional SEM images of other commercial high-silica FAUs were taken for comparison (Fig. S2b and c†). The commercial FAUs are known to possess a few mesopores, but there were much fewer voids observed as compared with the high-silica FAU obtained in this study.

In order to understand the formation mechanism of voids, it is essential to know more details on this synthetic process. Therefore, we investigated the recrystallization behaviour of high-silica FAU in the next section.

### Recrystallization process

**Fig. 4:**
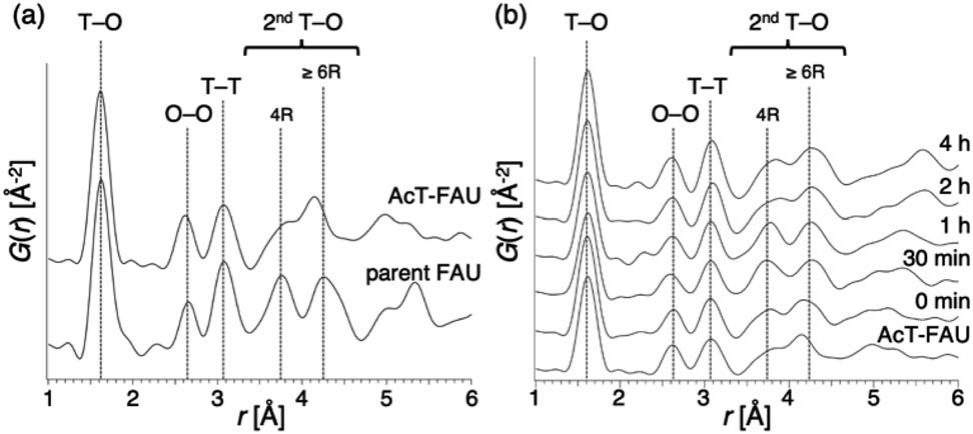
Atomic pair distribution function of various materials (a) parent FAU and acid treated FAU (AcT-FAU), and (b) products obtained from AcT-FAU at different heating times. The chemical composition of starting reactants is 1.0 SiO_2_ : 0.013 Al_2_O_3_ : 0.15 Na_2_O : 0.20 DMDMPOH : 5.0 H_2_O. The attribution of the peaks was based on previous studies.

To confirm the above discussion and clarify the formation process, we varied the NaOH content in the reactant and the synthesis time. The chemical composition of the starting reactant was 1.0 SiO_2_ : 0.013 Al_2_O_3_ : *x* Na_2_O : 0.20 DMDMPOH : 5.0 H_2_O (*x* = 0.050, 0.10, 0.15) and the heating time was 0–4 h. Furthermore, a stainless-steel tube reactor was used to understand the effect of synthesis time accurately. It transfers heat faster than a typical Teflon®-lined reactor due to the large surface area per unit volume, thereby resulting in a quicker temperature response in the reactor during the initial synthesis stage.^39,40^ The results of the products were summarized in Table S1.† In addition, XRD patterns of the products obtained at different synthesis times under various Na_2_O/SiO_2_ ratios are shown in Fig. S3–S5.† A decrease in NaOH, which was not only an inorganic structure-directing agent but also acts a mineralizer, slowed the crystallization of FAU zeolite, and sufficient crystallization did not occur at *x* = 0.05. The slow crystallization was probably due to both the lack of alkalinity to dissolve the amorphous aluminosilicate and the inadequate amount of Na^+^ cations as SDAs to form FAU. As far as XRD patterns are seen, in the condition that Na_2_O/SiO_2_ was 0.10, the crystallization of FAU proceeded moderately and completed after 2 h, and then FAU remained the same for 4 h (Fig. S4†). However, only a few amorphous matters were observed as shown in the SEM images (Fig. S6†). On the other hand, pure FAU was obtained after 30 min and pure AEI zeolites were obtained after 4 h when the Na_2_O/SiO_2_ was 0.15 (Fig. S5†). These results suggested that an increase in NaOH accelerated the recrystallization of FAU and the production rate of another phase, AEI. This difference in the products during the synthesis processes can also be confirmed from the SEM images (Fig. S6†). For a Na_2_O/SiO_2_ of 0.15, rectangular particles derived from AEI zeolites started to appear after 1 h, and only AEI crystals were confirmed at 4 h. In contrast, in the condition that Na_2_O/SiO_2_ was 0.10, there is no significant difference in the morphology of the samples over time, only FAU crystals were confirmed. Moreover, in order to clarify the influence of dealumination on the parent FAU and understand the changes in the short-range structure during the synthesis process, we performed a PDF analysis based on the HEXTS data of the products obtained at different synthesis times. Based on the obtained results, *G*(*r*)s were calculated from *S*(*Q*)s according to previous methods. The results are shown in Fig. 4 and S7.† The attribution of the peaks was based on the previous studies.^41,42^ As shown in Fig. 4a, the peak derived from the T–O bond observed at approximately 1.6 Å had shifted slightly to the left towards a short distance with an increase in the Si/Al ratio by dealumination. Additionally, for the peaks derived from the 2^nd^T–O observed at 3.6–4.5 Å, it was found that the peak at approximately 3.75 Å from 4R contained in the parent FAU had broadened significantly by dealumination and the peak at approximately 4.25 Å from 6R had also shifted. This suggests that the relatively large ring structure with 6R or more had been decomposed by dealumination and AcT-FAU had a large fraction of the 4R in the structure. This result is consistent with the result of the UV-Raman spectra (Fig. 1d). Comparing the products obtained at different heating times (Fig. 4b), the peaks derived from the 2^nd^T–O have shifted to the long-distance side as the synthesis process from AcT-FAU to high-silica FAU followed by high-silica FAU to AEI proceed. The respective peak positions corresponded to the simulated 2^nd^T–O distance derived from the 4 and 6Rs based on the atomic coordinate data of the FAU and AEI framework structures.^43^

**Fig. 5:**
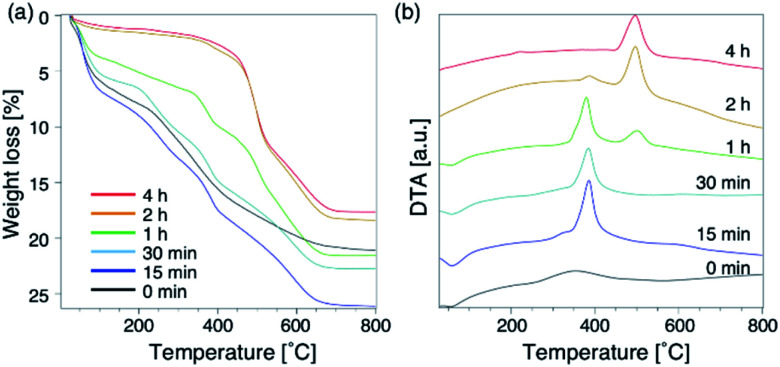
Thermogravimetry-differential thermal curves of products obtained at different synthesis times. (a) TGA, and (b) DTA. The chemical composition of starting reactants is 1.0 SiO_2_ : 0.013 Al_2_O_3_ : 0.15 Na_2_O : 0.20 DMDMPOH : 5.0 H_2_O.

To further understand the synthetic process, we analysed the composition of the solid phase in detail. For comparison, the products from the starting reactants with Na_2_O/SiO_2_ = 0.10 as well as Na_2_O/SiO_2_ = 0.15 were also analysed. First, to understand the role of the DMDMP^+^ cations during this synthesis process, the amount of organic compounds contained in the solid phase after different heating time was calculated based on the TG curves (Fig. 5a). As listed in Table S2,† the amount of organic matters contained in all samples for 15 min to 4 h was approximately 16 wt%, which was almost constant. This tendency was also observed for the Na_2_O/SiO_2_ of 0.10 (Table S3†). Considering the composition, these results mean that FAU contains approximately 2 DMDMP^+^ per *fau*-supercage and AEI zeolite contains approximately 1 DMDMP^+^ per *aei*-cage. The larger amount of DMDMP^+^ occluded in FAU is due to the silanol defects derived from the voids shown in Fig. 3, which were observed in ^29^Si MAS NMR (Fig. 2c). On the other hand, the amount of DMDMP^+^ contained in AEI is reasonable because the amount of DMDMP^+^ corresponds with that from previous studies of AEI zeolite synthesis with similar composition.^44–46^

Compared to the DTG curves (Fig. S8a†), it was confirmed that a peak of weight loss due to the combustion of organics shifted clearly to the high temperature region with the conversion from high-silica FAU to AEI. In addition, a peak derived from the heat of combustion observed in the DTA curves (Fig. 5b) also shifted similar to the DTG curves. These results suggest that the differences in the zeolite framework affects the thermal stability of DMDMP^+^. Actually, FAU has a supercage consisting of 4, 6, and 12Rs,^43^ whereas AEI zeolite has a cage structure consisting of 4, 6, and 8Rs.^43^ In other words, it can be said that the differences in the stability inside each cage affect the combustion temperature of DMDMP^+^.^47^ In fact, the DMDMP^+^ cation is known as one of typical OSDAs for AEI zeolite synthesis in previous studies,^17–20,44–46^ indicating that the DMDMP^+^ cation forms a more stable organic–inorganic complex with the AEI zeolite framework.^48,49^ Besides, a slight peak can be seen near 350 °C for 0–15 min in the DDTA curves (Fig. S8b†), which might be resulted from the combustion of DMDMP^+^ cations existing inside the amorphous aluminosilicates or on the crystal surfaces. It is assumed that the thermal stability of the DMDMP^+^ cation in the cages depends on the zeolite framework from these results.^47^

**Fig. 6:**
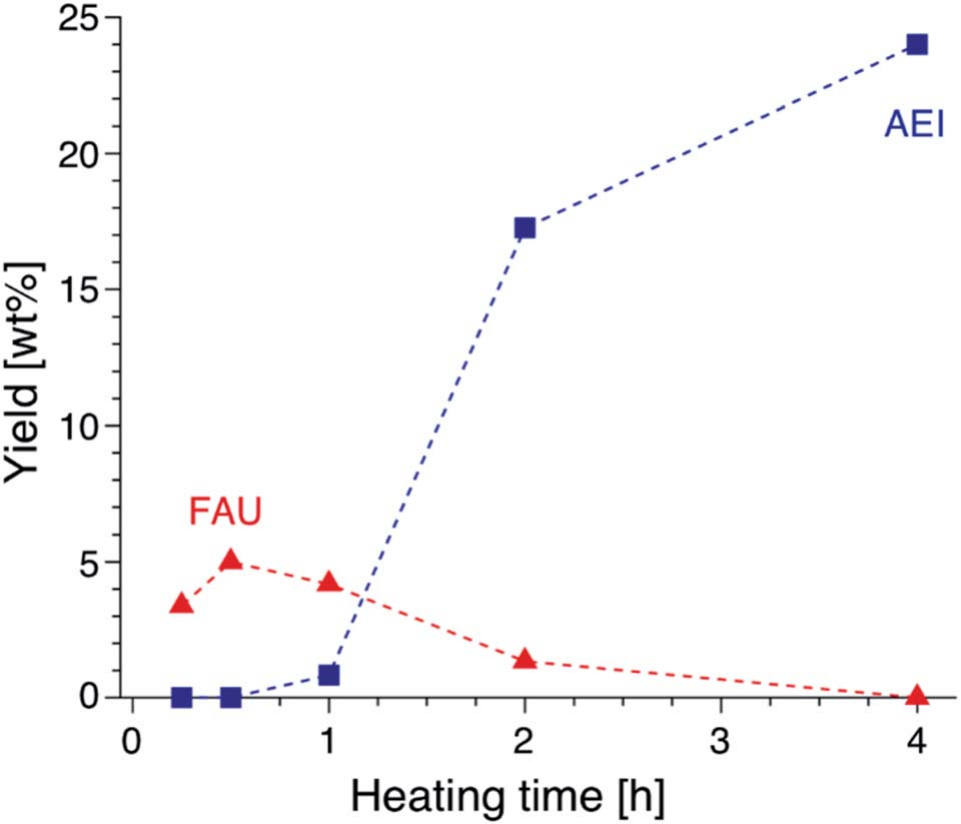
Yields of FAU and AEI obtained from AcT-FAU at different synthesis times. The proportion of each crystal phase was calculated from the relative crystallinity.

Focusing on the change in the composition of the solid phase by ICP-AES obtained at different synthesis times and the solid yield in addition to the above results of the TG analysis, a decrease in the Si/Al ratio is associated with the crystallization of high-silica FAU (Table S2†). Moreover, the Na/Al ratio increased with the crystallization time. Since there is almost no difference in the amount of DMDMP^+^ cations contained in the solid phase, high-silica FAU is formed while gradually incorporating Al atoms into the FAU zeolite framework. Then, the Na^+^ cations are also incorporated into ion-exchange sites generated by the incorporation of Al into the zeolite framework, which balances the charge of the additional Al atoms. Considering the crystallization process estimated from the composition change, it is speculated that the numerous voids in the high-silica FAU crystals shown in Fig. 3 were formed due to two main routes occurring at the same time, the crystallization with dissolution on the particle surface and the Si leaching inside the particle. Similar phenomena have been reported in previous studies,^50,51^ which suggested that the leached Si atoms contributed to the crystallization of a particle. Hence, it was considered that the same explanation could be applied to the formation of this high-silica FAU with large voids (Fig. S9†). In the subsequent zeolite conversion to AEI zeolite, the Si/Al ratio increased with the formation of AEI zeolite. Moreover, the Na/Al ratio decreased. Nonetheless, there was no significant difference in the proportion of DMDMP^+^ cations occluded in the solid phase. These facts strongly indicate that DMDMP^+^ cations play an important role in the charge compensation of the Al atoms associated with the crystallization of AEI zeolite. Focusing on the solid yield, the yield of high-silica FAU was very low immediately after heating, but reached 5.0 wt% with crystallization at 30 min. In addition, the solid yield increased significantly with the crystallization of AEI and the dissolution of FAU,^17–19^ and finally reached 24.0 wt% at 4 h (Fig. 6). Therefore, it is found that the high-silica FAU obtained in this study plays an intermediate role in the crystallization of AEI. Thus, the yield of high-silica FAU is low since it is formed due to the kinetic effect, whereas that of AEI is higher as more thermodynamically stable AEI zeolite is obtained at a prolonged synthesis time. This means that the AEI-DMDMP^+^ complex is more thermodynamically stable than the FAU-DMDMP^+^ one. Actually, this thermodynamic stability is consistent with the differences in the combustion temperature of the DMDMP^+^ cations in the TG curves.

**Fig. 7:**
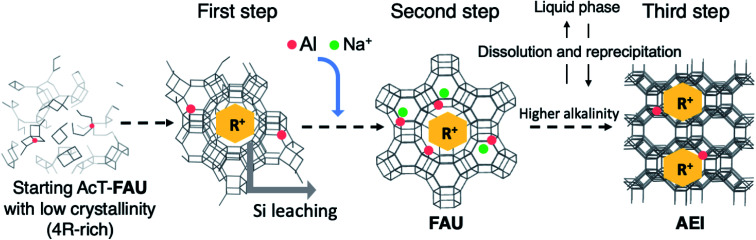
A proposed scheme of crystal growth process during the synthesis of AEI-type zeolite from acid treated FAU *via* recrystallization of high-silica FAU. R^+^ means an organic cation (DMDMP^+^). The first step represents the middle of the formation of high-silica FAU (at 0–15 min in Fig. S5†), the second step represents the completion of recrystallization of high-silica FAU (at 30 min in Fig. S5†), and the third step represents the formation of AEI-type zeolite (at 4 h in Fig. S5†).

To confirm the need for DMDMP^+^ in the formation of high-silica FAU, DMDMPOH-free syntheses were investigated. The reactant composition was 1.0 SiO_2_ : 0.013 Al_2_O_3_ : *x* Na_2_O : 0.0 DMDMPOH : 5.0 H_2_O (*x* = 0.15, 0.25). This mixture was heated at 180 °C for 1 h in a stainless-steel tube reactor. As shown in Fig. S10,† no peak derived from the FAU structure was observed by XRD patterns without the addition of DMDMPOH. Additionally, MOR zeolite was synthesized with the same amount of NaOH instead of DMDMPOH, which is similar with previous MOR synthesis from FAU.^52^ Considering that enough DMDMP^+^ cations were contained in the solid phase even after a short heating time, these results suggest that DMDMP^+^ cations contributes to the stabilization of the FAU zeolite framework from the early stages of high-silica FAU formation. From the above results, we proposed the synthesis scheme of AEI zeolite from AcT-FAU as schematically shown in Fig. 7. First, in the initial stage of heating, DMDMP^+^ cations and remained ordered aluminosilicates in AcT-FAU are combined to stabilize the FAU zeolite framework (at 0–15 min in Fig. S5†). Secondary, the Al atoms are incorporated into the FAU framework through heating. Finally, Na^+^ cations are also incorporated into the ion-exchange sites to balance the charge (at 30 min in Fig. S5†). Furthermore, Si leaching occurs simultaneously inside the particle, which is similar to the reaction scheme reported in previous studies.^50,51^ This reaction forms voids in the high-silica FAU with a Si/Al ratio of 6–8. Moreover, when NaOH is insufficient, the crystallization of FAU does not proceed. In addition, under a sufficient alkaline condition, nucleation and crystal growth of AEI zeolite occur (at 4 h in Fig. S5†). Therefore, high-silica FAU dissolves with the crystallization of AEI zeolite since it is also used as a Si and Al source.^18^ In this AEI zeolite, DMDMP^+^ contributes to the charge balance of the Al atoms.

## Conclusions

FAU-to-AEI interzeolite conversion was performed using AcT-FAU. During this synthesis, a relatively high-silica FAU with a Si/Al ratio of 6.1 was obtained for 2 h in the Teflon®-lined reactor sealed in a stainless-steel autoclave, which had large voids. The AcT-FAU prepared in this study had an ordered aluminosilicate structure with very low crystallinity evaluated by UV-Raman and HEXTS measurements. Furthermore, the roles of NaOH and DMDMPOH were clarified by detailed analysis in the synthesis process. In particular, the DMDMP^+^ cation has a positive impact on the crystallization and stabilization of FAU. To the best of our knowledge, there have been no reports of FAU synthesis using DMDMP^+^. It is expected that this finding can help the direct synthesis of high-silica FAU using amorphous matters. Also, investigating the specific synthesis process in this study will help to understand the role of organic structure-directing agents in the kinetical formation of products.

## Author contributions

The manuscript was written through contributions of all authors. All authors have given approval to the final version of the manuscript.

## Conflicts of interest

There are no conflicts to declare.

## Supplementary Material

RA-011-D1RA03150C-s001
