# Supplementary material for: Tracking the crystallization behavior of high-silica FAU during AEI-type zeolite synthesis using acid treated FAU-type zeolite
Source: RSC Adv. 2021 Jun 29;11(37):23082–9. doi: 10.1039/d1ra03150c (PMC9034275; doi:10.1039/d1ra03150c)
Supplement: RA-011-D1RA03150C-s001 [file RA-011-D1RA03150C-s001.pdf]

Electronic Supplementary Material (ESI) for RSC Advances.
This journal is © The Royal Society of Chemistry 2021

Electronic Supplementary Information

Tracking the Crystallization Behavior of High-Silica FAU During AEI-type Zeolite Synthesis Using Acid Treated FAU-type Zeolite

Yuki Sada, [a)] Anand Chokkalingam, [a)] Kenta Iyoki, [a)] Masato Yoshioka, [b)] Tomoya Ishikawa, [b)] Yusuke Naraki, [b)] Yutaka Yanaba, [c)] Hiroki Yamada, [d)] Koji Ohara, [d)] Tsuneji Sano, [a)] Tatsuya Okubo, [a)] Zhendong Liu, [a,e)*] and Toru Wakihara [a,e)**]

[a] *Department of Chemical System Engineering, The University of Tokyo, 7-3-1 Hongo, Bunkyo-ku, Tokyo 113-8656, Japan*

[b] *Inorganic Materials Research Laboratory, Tosoh Corporation, 4560 Kaiseicho, Shunan, Yamaguchi, 746-8501, Japan*

[c] *Institute of Industrial Science, The University of Tokyo, 4-6-1 Komaba, Meguro-ku, Tokyo 153-8505, Japan*

[d] *JASRI, 1-1-1 Kouto, Sayo-cho, Sayo-gun, Hyogo 679-5198, Japan*

[e] *Institute of Engineering Innovation, School of Engineering, The University of Tokyo, 2-11-16 Yayoi, Bunkyo-ku, Tokyo 113-8656, Japan*

[*] *liuzd@chemsys.t.u-tokyo.ac.jp*, [**] *wakihara@chemsys.t.u-tokyo.ac.jp*

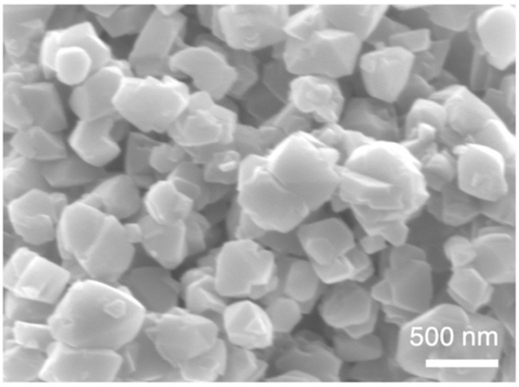

Fig. S1. SEM image of the parent FAU (HSZ-320HOA).

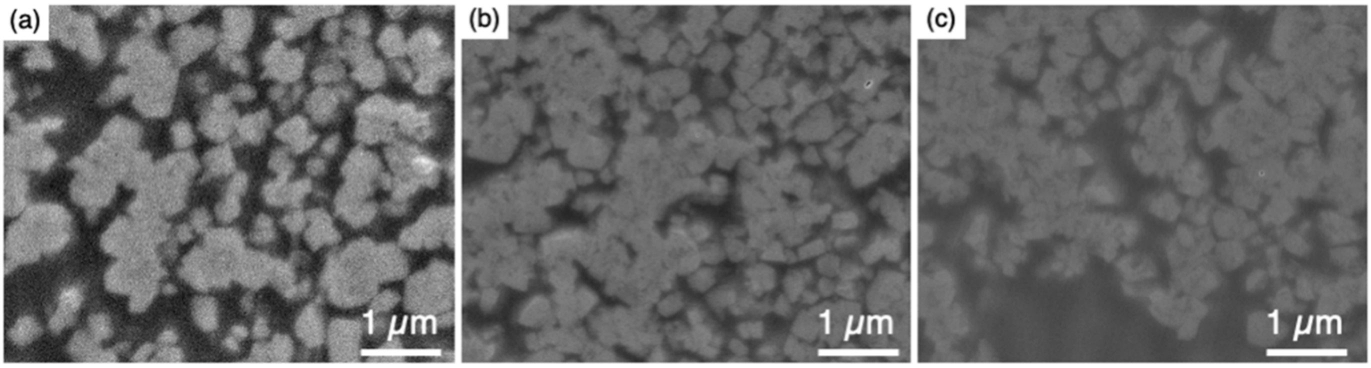

Fig. S2. SEM images of sliced commercial FAU. (a) HSZ-320HOA (parent FAU, Si/Al = 2.8), (b) HSZ-350HUA (Si/Al = 5.0), and (c) HSZ-360HUA (Si/Al = 7.0).

Table S1. Phases of products obtained at different heating times from starting reactants with various Na_2O/SiO_2 ratios.

x Na_2O/SiO_2	0 min	15 min	30 min	1 h	2 h	4 h
0.05	–	Amor. + FAU	Amor. + FAU	Amor. + FAU	Amor. + FAU	–
0.10	–	FAU	FAU	FAU	FAU	FAU
0.15	Amor. + FAU	FAU	FAU	FAU + AEI	AEI + FAU	AEI

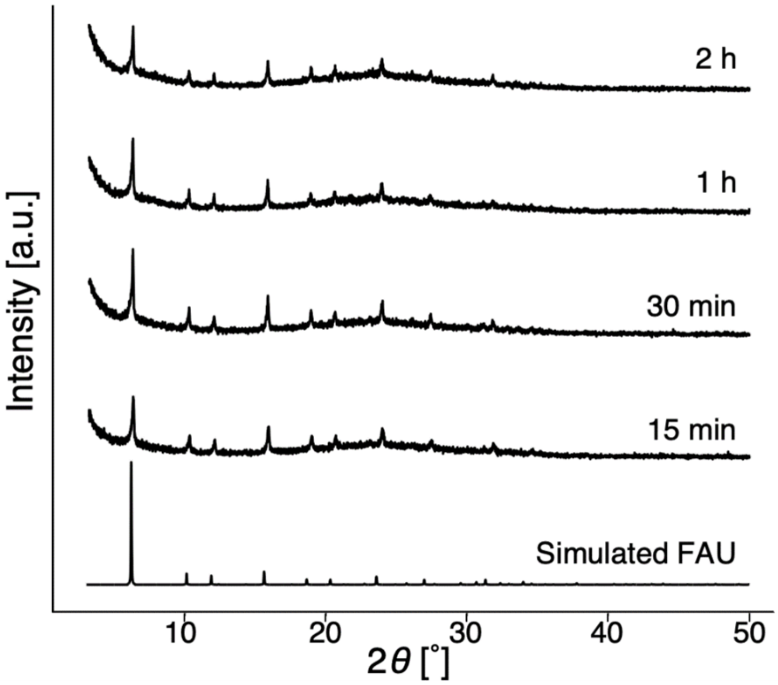

Fig. S3. XRD patterns of products obtained from acid treated FAU at different crystallization times from the starting reactant with a chemical composition of 1.0 SiO_2: 0.013 Al_2O_3: 0.05 Na_2O: 0.20 DMDMPOH: 5.0 H_2O.

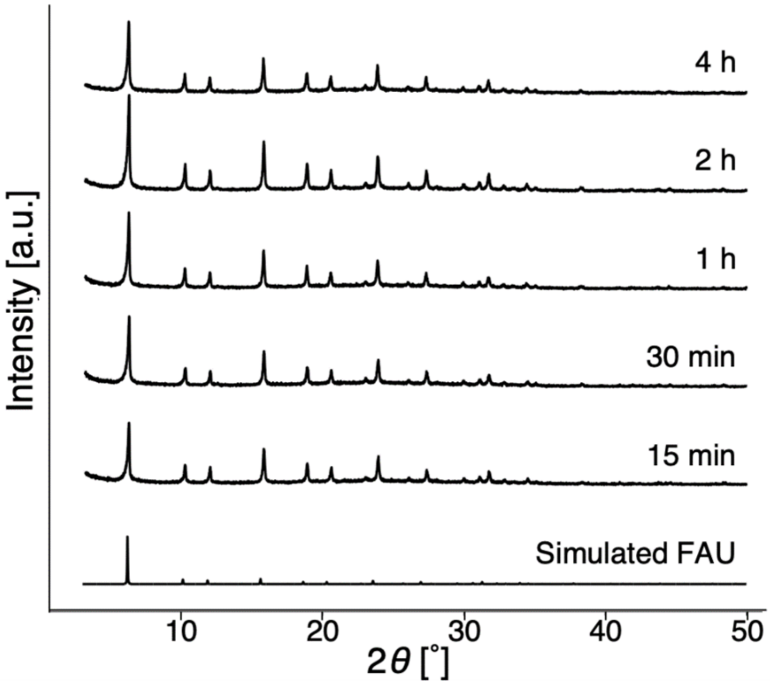

Fig. S4. XRD patterns of products obtained from acid treated FAU at different crystallization times from the starting reactant with a chemical composition of 1.0 SiO_2: 0.013 Al_2O_3: 0.10 Na_2O: 0.20 DMDMPOH: 5.0 H_2O.

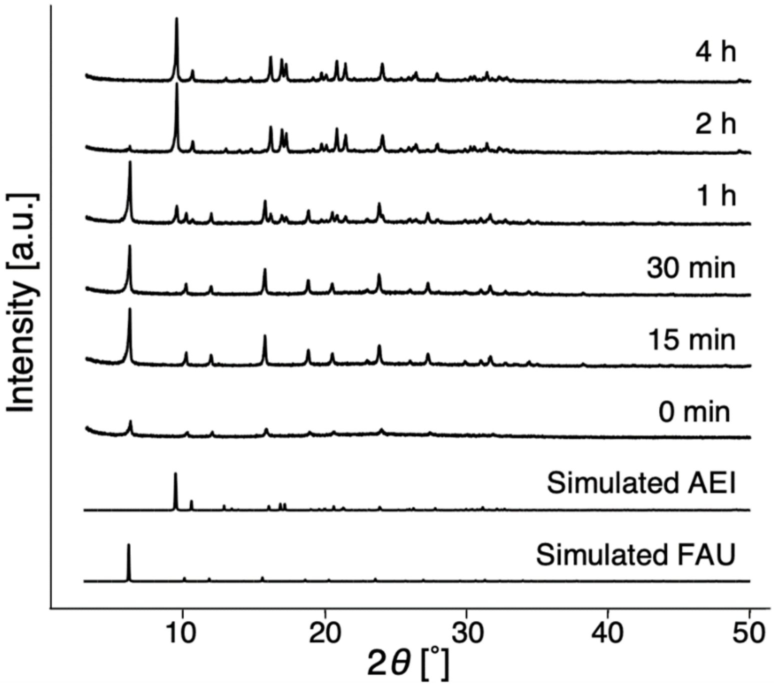

Fig. S5. XRD patterns of products obtained from acid treated FAU at different crystallization times from the starting reactant with a chemical composition of 1.0 SiO_2: 0.013 Al_2O_3: 0.15 Na_2O: 0.20 DMDMPOH: 5.0 H_2O.

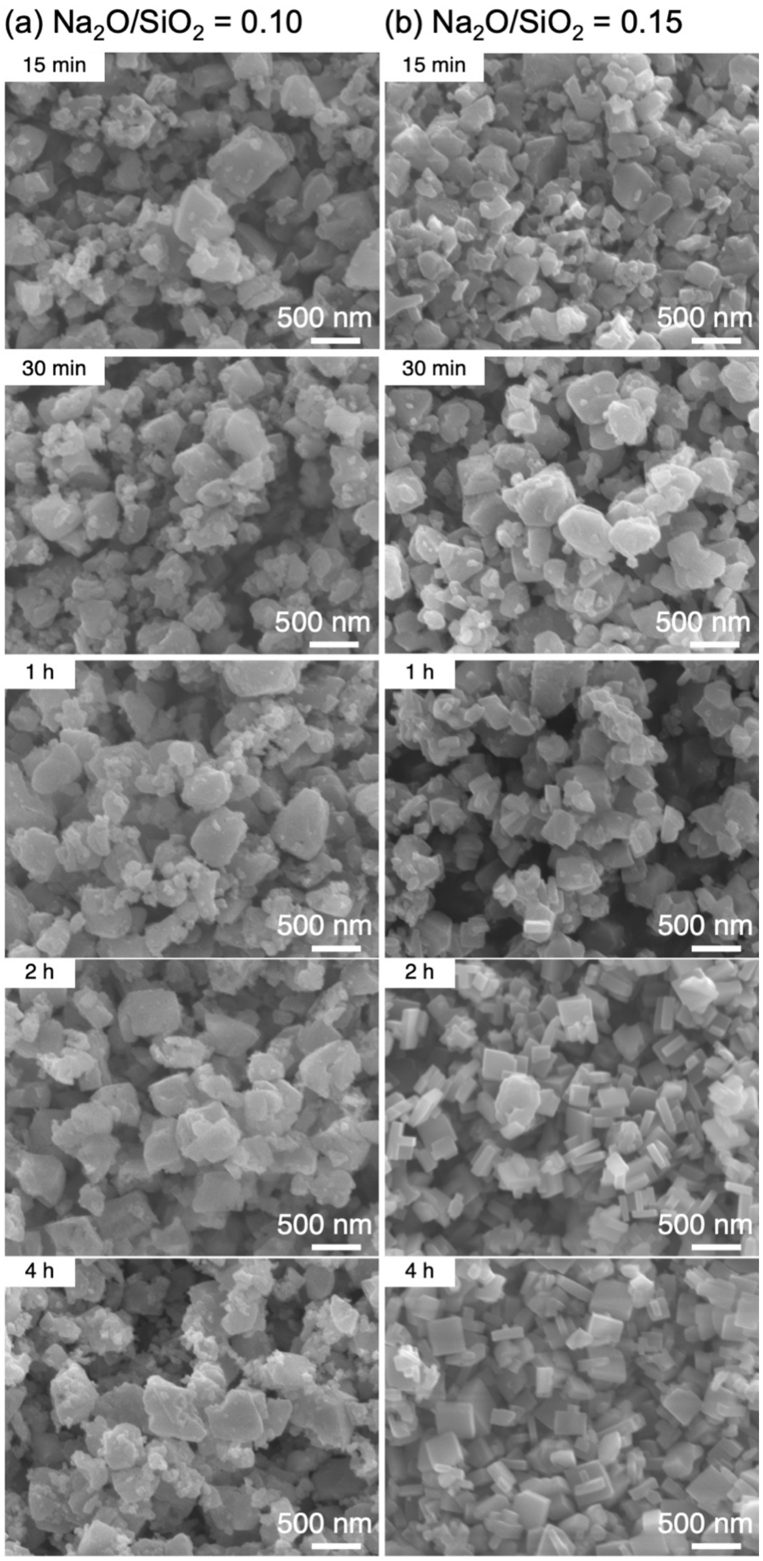

Fig. S6. SEM images of products obtained at different crystallization times from the starting reactants with Na_2O/SiO_2 ratios of (a) 0.10 and (b) 0.15.

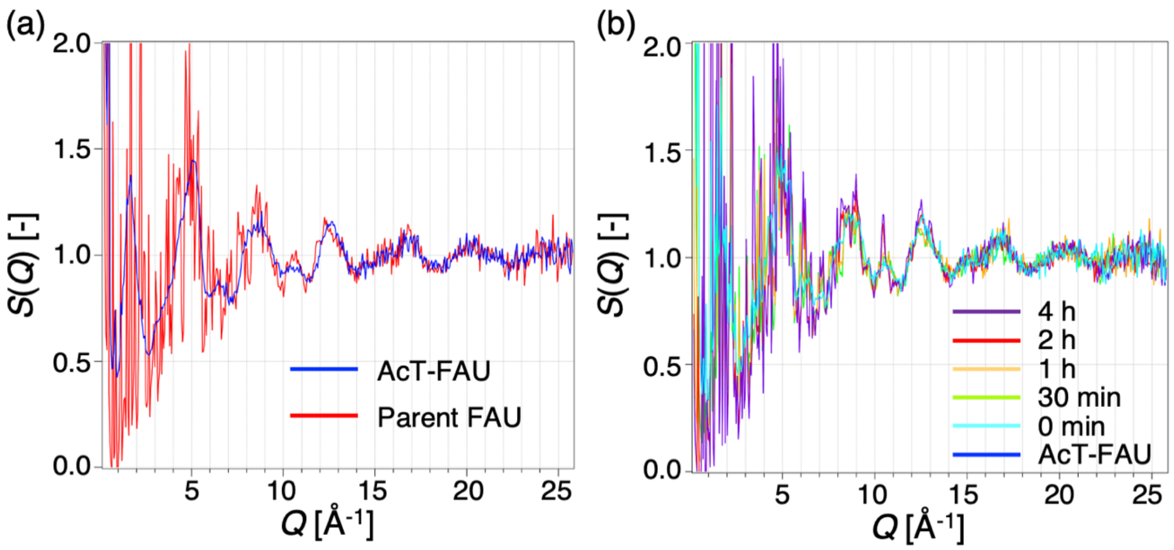

Fig. S7. *S*(*Q*)s of various materials, (a) parent FAU and AcT-FAU, and (b) products obtained from AcT-FAU at different crystallization times.

Table S2. Si/Al ratios, yields, and OSDA contents of products obtained at different crystallization times from the starting reactant with a chemical composition of 1.0 SiO_2: 0.013 Al_2O_3: 0.15 Na_2O: 0.20 DMDMPOH: 5.0 H_2O.

	15 min	30 min	1 h	2 h	4 h
Si/Al [–]	9.95	10.2	9.15	8.21	9.68
Solid yield [wt%]	3.39	2.19	3.40	4.00	5.60
Occluded OSDA [wt%]	16.5	15.3	16.0	15.1	15.5
Na/Al [–]	0.515	0.666	0.533	0.485	0.460

Table S3. Si/Al ratios, yields, and OSDA contents of products obtained at different crystallization times from the starting reactant with chemical composition a of 1.0 SiO_2: 0.013 Al_2O_3: 0.10 Na_2O: 0.20 DMDMPOH: 5.0 H_2O.

	0 min	15 min	30 min	1 h	2 h	4 h
Si/Al [–]	17.7	8.96	8.32	7.89	11.4	13.0
Solid yield [wt%]	9.40	3.21	5.00	5.01	18.6	24.0
Occluded OSDA [wt%]	12.7	16.3	15.3	16.1	16.7	16.4
Na/Al [–]	0.952	0.462	0.676	0.504	0.279	0.0146

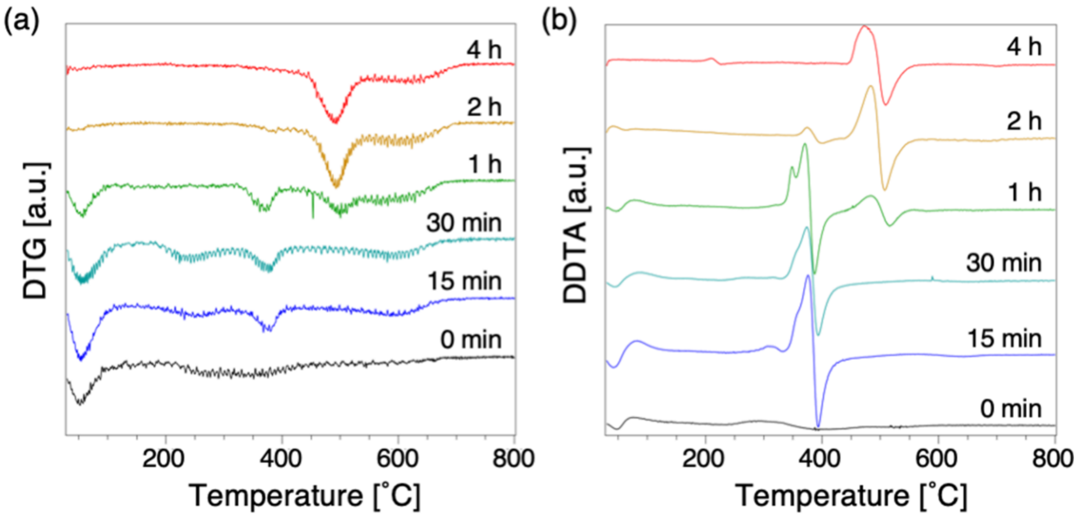

Fig. S8. Thermogravimetry-differential thermal curves of products obtained at different synthesis times. (a) DTG, and (b) DDTA. DTG and DDTA mean the time derivative of TGA and DTA, respectively.
The chemical composition of the starting reactant was 1.0 SiO_2: 0.013 Al_2O_3: 0.15 Na_2O: 0.20 DMDMPOH: 5.0 H_2O.

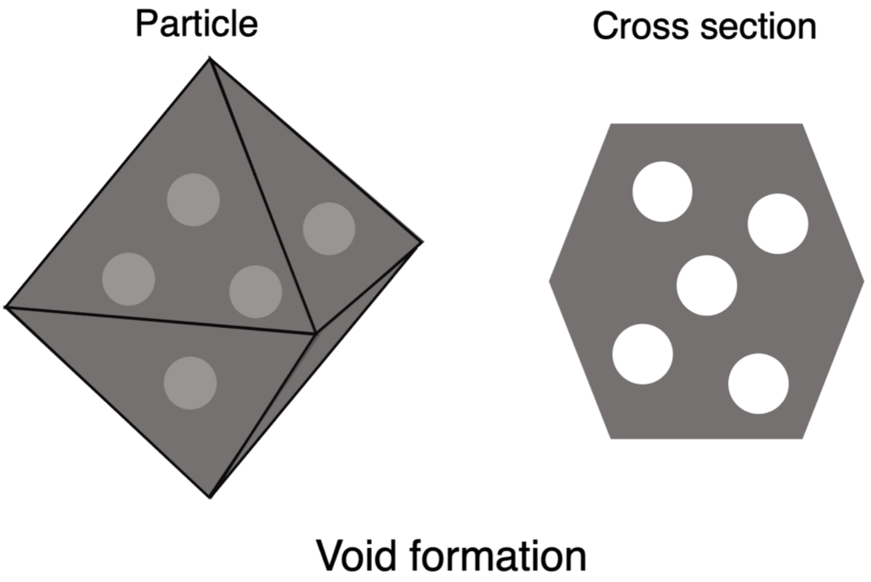

Fig. S9. The proposed model for the formed voids in high-silica FAU.

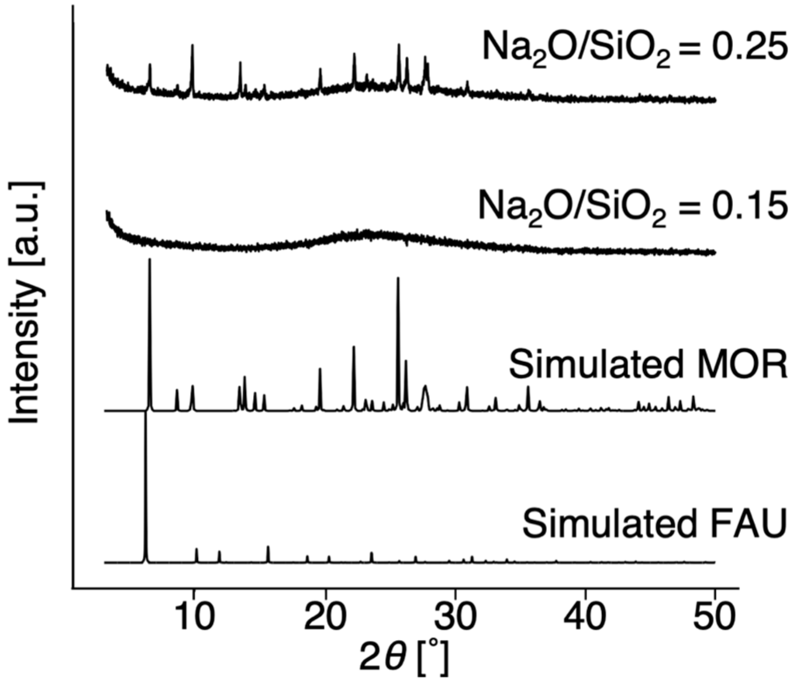

Fig. S10. XRD patterns of products obtained from the starting reactants with a chemical composition of 1.0 SiO_2: 0.013 Al_2O_3: x Na_2O: 0.0 DMDMPOH: 5.0 H_2O (x = 0.15, 0.25).
